# Metropolitan Hospital Sunday Fund

**Published:** 1897-10-30

**Authors:** 


### METROPOLITAN HOSPITAL SUNDAY FUND.

### The Out-patient Question.

At a well-attended meeting of the Council of the Metropolitan Hospital Sunday Fund, held at the Mansion House on Wednesday, 27th inst., Sir Sydney H. Waterlow, Bart., in the chair, the following special report of the Distribution Committee was adopted :—

"The Committee of Distribution having carefully reviewed all the evidence received by them during the last four or five years in reference to the out-patient departments of the several hospitals, and having recently obtained further information on this important subject, are of opinion that, where no person has been already appointed, efforts should be made to induce the governing bodies of hospitals to appoint efficient persons to make inquiries respecting patients attending out-patient departments, with the view of preventing any abuse, and that the Council of the Hospital Sunday Fund should exercise the greatest caution before interfering with the internal management of hospitals."

On the motion of the Chairman, seconded by Sir W. H. Broadbent, Bart., the following resolution was carried unanimously :—

"That this Council recommend that at the next meeting of the constituents in December the following clause be added to the Laws of the Constitution: That where any hospital having an out-patient department has appointed a special and efficient officer for detecting any abuses in that department, it shall be regarded by the Distribution Committee as an important factor in determining the merits."

A further report of the proceedings will be given in our next issue.

